# Capacity and Allocation across Sensory and Short-Term Memories

**DOI:** 10.3390/vision6010015

**Published:** 2022-03-01

**Authors:** Shaoying Wang, Srimant P. Tripathy, Haluk Öğmen

**Affiliations:** 1 Department of Electrical & Computer Engineering, University of Denver, Denver, CO 80210, USA; shaoying.wang@du.edu; 2 School of Optometry and Vision Science, Faculty of Life Sciences, University of Bradford, Bradford BD7 1DP, UK; s.p.tripathy@bradford.ac.uk

**Keywords:** sensory memory, short-term memory, attention, mixture model

## Abstract

Human memory consists of sensory memory (SM), short-term memory (STM), and long-term memory (LTM). SM enables a large capacity, but decays rapidly. STM has limited capacity, but lasts longer. The traditional view of these memory systems resembles a leaky hourglass, the large top and bottom portions representing the large capacities of SM and LTM, whereas the narrow portion in the middle represents the limited capacity of STM. The “leak” in the top part of the hourglass depicts the rapid decay of the contents of SM. However, recently, it was shown that major bottlenecks for motion processing exist prior to STM, and the “leaky hourglass” model was replaced by a “leaky flask” model with a narrower top part to capture bottlenecks prior to STM. The leaky flask model was based on data from one study, and the first goal of the current paper was to test if the leaky flask model would generalize by using a different set of data. The second goal of the paper was to explore various block diagram models for memory systems and determine the one best supported by the data. We expressed these block diagram models in terms of statistical mixture models and, by using the Bayesian information criterion (BIC), found that a model with four components, viz., SM, attention, STM, and guessing, provided the best fit to our data. In summary, we generalized previous findings about early qualitative and quantitative bottlenecks, as expressed in the leaky flask model and showed that a four-process model can provide a good explanation for how visual information is processed and stored in memory.

## 1. Introduction

**Figure 1:**
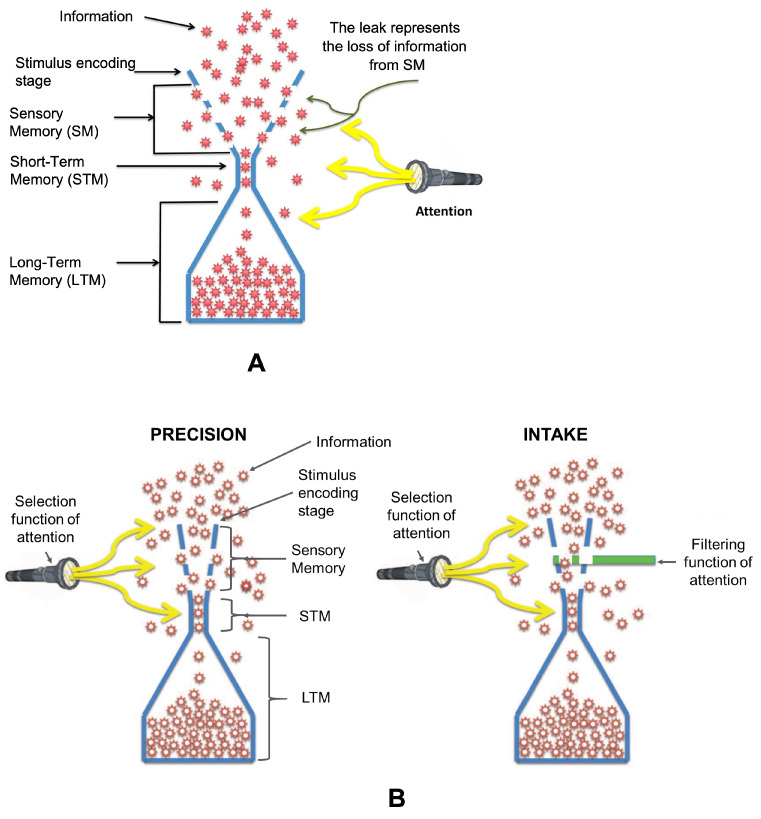
The leaky hourglass model (**A**) and the leaky flask model (**B**). In the leaky hourglass model, the large SM capacity is shown by the width of the hourglass at the top. The leak through the holes represents the fast decay of SM. The bottleneck is at the STM stage, which is followed by a large capacity LTM. In the leaky flask model, the top part is narrower, indicating information processing limits prior to STM. There are two leaky flasks, one representing the quality of information (precision) and the other the quantity of information (intake). The main bottleneck for the quality of information is at the stimulus encoding stage rather than the memory stage. The bottleneck for the quantity of information is more distributed, spreading from the encoding stage to the memory stage. Figure from Öğmen et al. [21].

It is a challenge to understand how the continuous stream of information available to the visual system is processed in sensory memory (SM), short-term memory (STM), and long-term memory (LTM). SM has a large capacity, but decays rapidly in a few hundred milliseconds [1,2,3,4]. STM lasts longer with a limited capacity [5,6,7,8,9,10,11,12,13,14,15,16,17,18], constituting a bottleneck in information flow [9,10,19,20]. This classical view of capacities and bottlenecks can be captured by the leaky hourglass model depicted in Figure 1A. The red items in the figure represent information, which is first encoded with a large capacity encoding stage and stored in SM. Whereas SM’s capacity is large, as depicted by the broad top of the hourglass, information is lost at a rapid pace, which in turn is shown as a leak from SM. The narrow neck of the hourglass in the middle represents the limited capacity of STM. In classical models of memory, a single temporal decay process is used to explain the temporal dependence of the quality and quantity of information in memory. As the representations of items decay in time, the quality of information becomes degraded and the number of items available for storage becomes smaller.

However, a recent study showed three major departures from these models: First, unlike the notion that the bottleneck resides in STM, major bottlenecks were found prior to STM. Second, the temporal evolutions of the quality and quantity of information are different: only a small decay in the quality of information was observed over a time interval during which a substantial decay in the quantity of information occurred (Figure 9 in [21]). Third, attention interacts differently with the quality and quantity of information: whereas the selection function of attention modulates both the quality and quantity of information, the filtering function of attention influences only the quantity of information [21]. Hence, the leaky hourglass model has been replaced by two leaky flasks, as shown in Figure 1B [21]. By changing the hourglass to a flask, the narrower top part illustrates capacity limits prior to STM. By using two “flasks”, the model highlights the different dynamics of quality and quantity, as well as the different ways attention interacts with these two aspects of memory storage. How these schematic models are implemented in the brain needs further investigation. The two leaky flasks model does not imply that there are two separate stores, one for the quality and one for the quantity of information. It could still be one “store”, but two processes. As an example, the computations of a visual object’s boundaries and surface follow different processes, but their outcomes are combined to produce a unified object representation.

The main bottleneck for the quality of information (precision) is at the stimulus encoding stage rather than the memory stage (Figure 1B). The bottleneck for the quantity of information (intake) is more distributed, spreading from the encoding stage to the memory stage (Figure 1B). The leaky flask model was proposed based on the results of one set of experiments. In this work, our first goal was to test this model further by using an alternative dataset from a different experiment [22].

STM has a limited capacity [5,6,7,8,13,15,16,17,18]. One approach to modeling STM is to view it as a system with a fixed discrete capacity [8,18,23,24,25]. In the fixed discrete capacity model, the number of items that can be remembered is fixed. The quality of memory can be improved only if the memory load is below the capacity [18,26,27]. When the items available for processing exceed the fixed capacity, the excess items are discarded [9,12,28,29]. The other approach is to model STM as a continuous resource [8,17,30,31]. In this approach, there is no limit to the number of items that can be remembered, and the limited memory resources are shared across all the processed items [8,17,23]. There is also a dynamic resource model of STM [8,32], which proposes that STM has limited resources, which can be allocated flexibly, resulting in items being stored with variable fidelity [5,8,17,23,32].

A recent study also proposed that the precision of STM is not fixed and modeled the precision of STM as having a variable component [26,33,34,35]. In the study of Fougnie et al. [26], subjects were asked to use a color-wheel to report the colors of a set of dots following a brief cue delay after their presentation. The distribution of the recalled colors was found to be more peaked than a normal distribution, and a variable precision model could capture the data better than a fixed precision model. They also showed, within a single trial, that the variability between items was different. Furthermore, the memory qualities across items was not influenced by each other [26]. The variable precision model and several alternative models were tested in several studies [34,35]. They found that the variable precision model can capture the data better. However, the origin of the variability is not yet clear [30,33,34,35]. Here, we investigated several different block diagram models of memory to relate the statistical components of the mixture model to a combination of information processing and storage processes.

## 2. Materials and Methods

The current paper modeled some of the data presented in the psychophysical study by Tripathy and Öğmen [22]. The experiments in the original study were conducted with the approval of the Research Ethics Committee of the University of Bradford, and all participants provided written informed consent prior to their participation. The current study did not involve the participation of human subjects and modeled existing data from the study published earlier [22].

**Figure 2:**
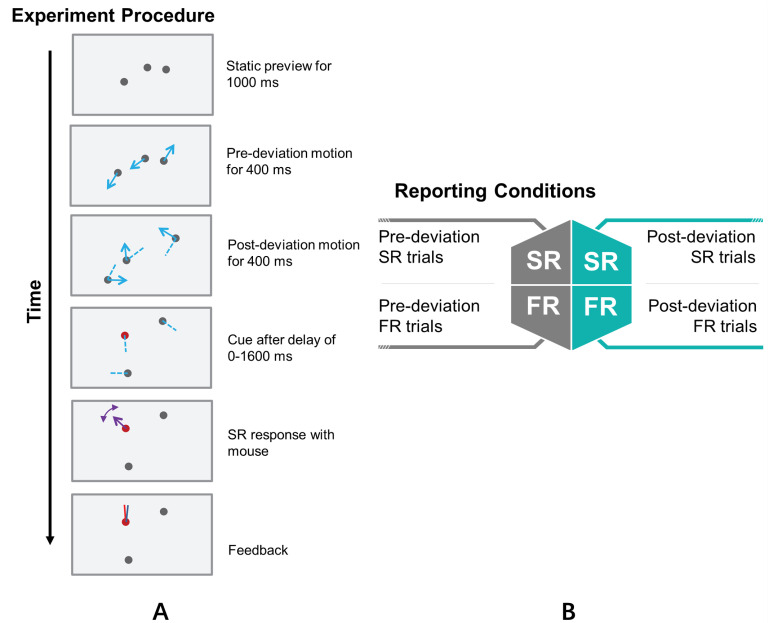
Experimental procedure and reporting conditions. (**A**) Sample trial from Tripathy and Öğmen, 2018, for a stimulus consisting of three disks and requiring the report of the post-deviation direction of motion of a single disk (SR). A mouse click from the observer initiated the trial. Three randomly positioned disks appeared on the screen and, after a delay of 1000 ms, moved in different directions for 400 ms. The disks then randomly changed directions by 30–180° and then moved along the new directions for another 400 ms before disappearing. Blue solid arrows show the trajectories in the latter time interval, and dashed blue lines show their previous trajectories. After the cue delay (0–1600 ms), the disks reappeared at the point of disappearance, and one of the disks was marked in red for reporting. (**B**) The four reporting conditions were: single report (SR) and full report (FR) for the pre-deviation trajectories and SR and FR for the post-deviation trajectories. (The arrows and dashed lines plotted in the stimulus figure are for illustration only; they were not shown during the experiments.)

The details of the stimuli (Figure 2A) that were used and the behavioral experiments that were conducted in the original study can be found in Tripathy and Öğmen [22] and, for the sake of continuity, are summarized below.

In the modified multiple object tracking (MOT) paradigm [21,22,36,37,38,39,40,41,42], the stimuli consisted of 1, 2, 3, or 4 disks moving in random directions, along bilinear trajectories, i.e., the trajectories of the dots were straight lines, except for one change in direction or deviation that occurred exactly at the mid-point of each trajectory (see Figure 2A). The start of motion, the end of motion, and the change in direction for the different disks in a trial were synchronized. The change in direction that occurred mid-way through the trajectories was either clockwise or anti-clockwise, with equal probability, and the magnitude of the change angle was randomly selected from a uniform distribution ranging from 30–180°; the deviations of the different discs in a trial were uncorrelated. The large and synchronized deviations in the trajectories in a trial were intended to produce an event boundary between the pre-deviation and post-deviation event segments. The trajectories of the disks were constrained so that no part of the trajectories extended beyond the edge of the computer screen. The disks in the stimuli were grey (7.0 cd/m${}^{2}$ on a white background of luminance 64.9 cd/m${}^{2}$), had a diameter of 1${}^{\circ }$, and moved at a speed of 5${}^{\circ }$/s for a total duration of 800 ms.

Observers were cued to report the direction(s) of motion in either the pre-deviation event segment or the post-deviation event segment. Additionally, the report could be the direction of motion of a single randomly selected disk (*single report* (SR)) or the directions of motion of all of the disks (*full report* (FR)) within an event segment. For FR, the order of reporting the directions was at the discretion of the subject. In summary, the type of report belonged to one of four categories: pre-deviation SR, post-deviation SR, pre-deviation FR, or post-deviation FR (see Figure 2B). In Experiment 1, the set size was varied—in each trial, one, two, three, or four disks were presented, with the set size being blocked; the set size remained fixed within a block and was randomly varied between blocks. The different report types were randomly interleaved within a block, and in each trial, a cue, presented immediately after the termination of the motion of the disks (cue delay = 0 ms) informed the observer of the report type expected. In Experiment 2, the set size was fixed at three, and there was a variable delay between the termination of disk motion and the presentation of the cue; the cue delay was fixed within a block and varied between blocks (the cue delay was 0, 100, 200, 400, 800, or 1600 ms). Using the mouse, observers reported, according to the cue presented, one (SR) or all (FR) of the directions of motion, in either the pre-deviation event segment or the post-deviation event segment. The computer recorded, for each direction of motion reported, the error associated with the report, as well as the response time. Following the observer’s response in each trial, feedback was provided as to the actual direction(s) of motion and the reported direction(s) of motion (see Figure 2A).

### 2.1. Models

In the current study, we modeled some of the data from the Tripathy and Öğmen [22] study using variations of the modeling approach presented in our earlier study [21]. Öğmen et al. [21] presented psychophysical data for errors in reporting the directions of motion of several disks in simultaneous motion along linear trajectories (see also [43]) and modeled these errors using a variety of approaches. Here, we used the insights obtained from the modeling in Öğmen et al. [21] to model the errors in the reported directions of motion in the Tripathy and Öğmen [22] study. The details of the modeling in the original study and the adaptations in the current study for modeling the Tripathy and Öğmen [22] dataset are described below.

#### 2.1.1. One Gaussian + Uniform Mixture Model

In the different experiments, our dependent measure in each trial was the *error* angle (ε) between the actual direction of motion of a disk and its reported direction of motion. The error angle was converted using the equation below to obtain the transformed performance (TP) [21,36,37,43]:

(1) TP=1−|ε|⁄180

TP is a bounded dimensionless quantity similar to a probability measure, with values of 1 and 0.5 corresponding to perfect- and chance-level performances, respectively. Thus, as in Shooner et al. [43] and in Öğmen et al. [21], TP was our preferred direct response measure for reporting psychophysical performance because its probability-like properties facilitate visualization and mathematical manipulation.

Traditionally, other indirect measures of performance have been utilized for reporting psychophysical performance. One such measure of performance is capacity, i.e., the number of items that can be processed or stored in memory [9,38]. However, it is well known that the precision of storage is influenced by the number of items that need to be stored [26,41] and the number of items that can be processed is influenced by the precision required [26,39]. Therefore, a proper characterization of performance requires that its quantitative measure (i.e., how much information is stored) and its qualitative measure (i.e., the precision with which the information is stored) be estimated [18,21,23].

In order to estimate qualitative and quantitative measures of performance, Öğmen et al. [21] modeled the distribution of errors for reporting the direction of motion of the disks with the following descriptive statistical models: Gaussian; Gaussian + uniform; Gaussian + uniform + misbinding. The Gaussian distribution represented processing or storage of information in memory; the uniform distribution represented guessing for information that is not in memory; the misbinding term represented reporting the direction of motion of an item different from the one that was to be reported. The model that best described the distribution of errors was the Gaussian + uniform model outlined below.

Öğmen et al. [21] found that the one Gaussian + uniform mixture model best described the distribution of the reported errors’ *PDF*(ε):

(2) PDF(ε)=ωG(ε;μ,σ)+(1−ω)U(a,b)

The mean (μ) of the Gaussian distribution represents the accuracy of the reported directions of motion, and the reciprocal of the standard deviation (σ) represents the precision of encoding the directions. The weight of the Gaussian (ω) is the proportion of trials on which the subject responds using stored target motion information, which is a relative measure for the intake of encoding, i.e., the proportion of presented items that are stored or processed for a given stimulus. The precision and the intake represent the qualitative and quantitative aspects of performance. The uniform distribution (*U*) over the interval (*a* = −180${}^{\circ }$, *b* = 180${}^{\circ }$) represents guessing the direction of motion of the target when no stored motion information is available. The weight of the uniform distribution (1−ω) represents the proportion of guesses across trials. A nonlinear optimization routine was created using the MATLAB fminsearch() function to estimate the parameters of this model. Model outputs were calculated by using estimated parameters and compared to behavioral data. This nonlinear optimization routine was used to find the best-fitting model for the distribution of behavioral data in each experimental condition.

We adapted the Gaussian + uniform mixture model from Öğmen et al. [21] to model our errors in reporting motion direction. The model was applied separately to the trajectories in the pre-deviation event segment and in the post-deviation event segment. For each of the two event segments, estimates of the two parameters, precision and intake, were obtained as functions of the set size in Experiment 1 and as a function of the cue delay in Experiment 2. As we were able to make specific predictions for the single report condition (SR) and on the first reported item in the full report condition (denoted by FR1) for the post-deviation event segment, we focused our modeling on those two conditions, though other reporting conditions were also modeled.

#### 2.1.2. Multiple Gaussians Models

It is well known that visual memory consists of multiple stages, with different intakes and precisions. For example, differences in quantitative measures of performance between sensory and visual STM have been well documented [8,18,23,25,44] and differences in qualitative measures have been less investigated. The variable precision model indicates that the STM of stimuli would not be a fixed precision with a single Gaussian distribution, but a variable precision with an infinite mixture of Gaussian distributions (variable precision models). Here, we used multiple Gaussians + uniform mixture models to determine the origins of variability in precision. The data were fit by the one, two, three, four, and five Gaussian(s) + uniform mixture models. Detailed fitting method could be found in Appendix A.

**Figure 3:**
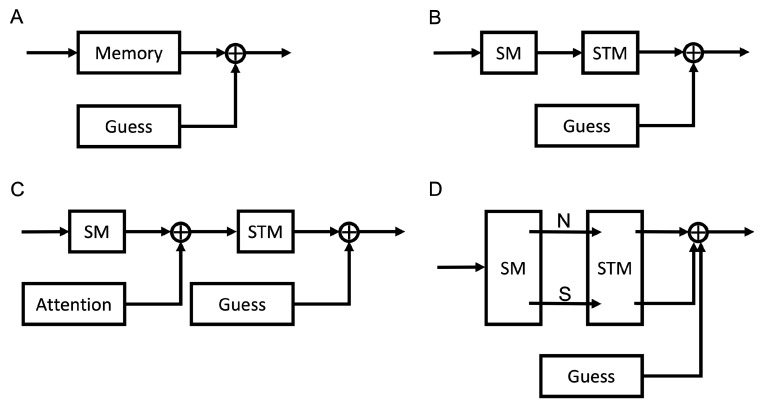
Block diagrams of the different models investigated. (**A**) Model A, one Gaussian + uniform mixture model. (**B**) Model B, with memory represented as a concatenation of SM and STM. This is still a one Gaussian + uniform mixture model. (**C**) Model C has attention added to the model, with the SM block being pre-attentive. The resulting model is a two Gaussians + uniform mixture model. (**D**) Model D divides the SM and STM stages into two parallel sections. This also represents a two Gaussians + uniform mixture model.

*Model A*: In the previous section, one of the models we used to analyze the bottlenecks of motion processing was the one Gaussian mixture model [21]. The one Gaussian mixture model could roughly capture the data pertaining to the reported directions of motion of multiple moving objects by lumping the multiple memory stages in visual processing into one single memory stage, as shown in Model A in Figure 3A.

(3) $$S_{R}\left(\varepsilon \right)=\omega G_{Memory}\left(\varepsilon ;\mu_{Memory},\sigma_{Memory}^{2}\right)+\left(1-\omega \right)U\left(a,b\right)$$

Visual task processing typically involves multiple stages, including stimulus encoding, storage of the stimulus representation in SM, STM, and/or LTM, etc. Here, we propose several possible detailed models linking the different mechanisms involved in visual processing (see Figure 3), modeling the processing within each mechanism as a Gaussian distribution for information available in memory and as a uniform distribution when information is unavailable in memory. Most processes were presumed to have a fixed mean (μ) and a fixed standard deviation (or SD (σ)) that do not change over time for the durations investigated here. The exception was SM, which involves information decaying rapidly over time, resulting in the standard deviation for reporting motion direction increasing with cue delay over durations of up to a second or so.

The standard deviation of the error distribution is represented by $\sigma_{process}$, where the process could represent different stages in visual processing such as: information encoding, STM, etc. The standard deviation for a rapidly decaying SM is represented by $\sigma_{SM}\left(t\right)$, indicating that this parameter varies as a function of time *t*.

*Model B*: In the classic modal model [45,46], the single block representing memory processes in Model A was replaced by two sequential blocks representing SM and STM (Figure 3B). The signal (S) available to subsequent levels of processing has the equation:

(4) $$S_{R}\left(\varepsilon \right)=\omega G_{SM}\left({\varepsilon ;\mu }_{SM},{\sigma_{SM}}^{2}\left(t\right)\right)\;*\;G_{STM}\left(\varepsilon ;\mu_{STM},{\sigma_{STM}}^{2}\right)+\left(1-\omega \right)U\left(a,b\right)$$

where * represents the convolution operation. The convolution of two Gaussians is also a Gaussian with a mean and variance equal to the sum of the two means and variances, respectively. (Here, we derived the models for the general case assuming Gaussianity holds. In cases where Gaussianity is violated, e.g., when variables are defined on finite intervals causing the “clipping” at the ends of the interval, this becomes an approximation depending on the amount of clipping.) Using this simplification, this equation can be rewritten as:

(5) $$S_{R}\left(\varepsilon \right)=\omega G_{Memory}\left(\varepsilon ;\mu_{SM}+\mu_{STM},{\sigma_{SM}}^{2}\left(t\right)+{\sigma_{STM}}^{2}\right)+\left(1-\omega \right)U\left(a,b\right)$$

This is still a one Gaussian + uniform model.

*Model C*: Attention plays an important role in transferring information into STM. Its effects have been modeled as selective signal enhancement, distractor exclusion, and internal noise reduction [31,35,47,48,49,50,51,52,53,54,55,56,57]. In addition, Emrich et al. [35] showed that attention could mediate STM allocation flexibly. They reported that changes in spatial attention, rather than memory load accounted better for memory performance. Attended regions can have inhibitory surrounds [58,59], and crowding is also known to occur when distractor items approach attended items [60,61]. In spite of these characteristics of attention, we modeled the effects of attention as a single Gaussian distribution because we modeled behavioral data that were averaged across many trials. The central limit theorem predicts that the effects of attention across trials could be captured by a Gaussian distribution. From the statistical modeling perspective, adding an attention component that modulates the output of SM gives us Model C (Figure 3C).

(6) $$\begin{array}{c} S_{R}\left(\varepsilon \right)=\omega_{1}\left(\omega_{2}G_{SM}\left({\varepsilon ;\mu }_{SM},{\sigma_{SM}}^{2}\left(t\right)\right)+{\left(1-\omega_{2}\right)G}_{Attention}\left({\varepsilon ;\mu }_{Attention},{\sigma_{Attention}}^{2}\right)\right)\\ {}*\;STM\left(\varepsilon ;\mu_{STM},{\sigma_{STM}}^{2}\right)+\left(1-\omega_{1}\right)U\left(a,b\right) \end{array}$$

By simplifying, we obtain:

(7) $$\begin{array}{c} S_{R}\left(\varepsilon \right)=\omega_{1}\omega_{2}G_{SM}\left({\varepsilon ;\mu }_{SM},{\sigma_{SM}}^{2}\left(t\right)\right)\;*\;G_{STM}\left({\varepsilon ;\mu }_{STM},{\sigma_{STM}}^{2}\right)+\\ \;\omega_{1}{\left(1-\omega_{2}\right)G}_{Attention}\left(\varepsilon ;\mu_{Attention},{\sigma_{Attention}}^{2}\right)\;*\;\\ \;G_{STM}\left(\varepsilon ;\mu_{STM},{\sigma_{STM}}^{2}\right)+\left(1-\omega_{1}\right)U\left(a,b\right) \end{array}$$

(8) $$\begin{array}{cc} S_{R}\left(\varepsilon \right)= & \omega_{1}\omega_{2}G_{1}\left({\varepsilon ;\mu }_{SM}+\mu_{STM},{\sigma_{SM}}^{2}\left(t\right)+{\sigma_{STM}}^{2}\right)+\\ & \omega_{1}{\left(1-\omega_{2}\right)G}_{2}\left(\varepsilon ;\mu_{Attention}+\mu_{STM},{\sigma_{Attention}}^{2}+{\sigma_{STM}}^{2}\right)+\left(1-\omega_{1}\right)U\left(a,b\right) \end{array}$$

The result is a two Gaussians + uniform mixture model. Information in STM can be retained for several seconds, and this means the precision (inverse of the standard deviation) should hold as a constant for different cue delays.

Gegenfurtner and Sperling [62] showed that there are two kinds of information transfer from iconic memory to STM: nonselective transfer (where it is not known which items are of interest to perform the task) and selective transfer (where the items of interest are known, say due to cueing). From the onset of the stimulus display until the onset of the cue, subjects carry out unselective transfer and switch to selective transfer once the cue informs items of interest. Transfer of information to STM ensures more stable storage (its precision does not vary with time over the duration of a trial) relative to information that remains in SM (its precision is a rapidly decaying function of time). For the FR condition, the observer can start by reporting any item already stored in STM. Typically, the observer reports the best remembered item, which is the first item transferred into STM, because this item is the one that suffered the least from SM decay and maintained in STM close to its original encoding level. Hence, we predicted the first item of the full report (FR1) to depend very little on cue delay. Therefore, for FR1, we can predict that at least one of the two standard deviation parameters in the two Gaussians + uniform mixture model will not vary with the cue delay. On the other hand, for the single report condition, the cued disk was randomly selected and could be one that was not in STM (in particular when the set size increased). In this case, subjects must transfer the information from SM to STM for final reporting. As a result, in this case, the report will be a function of cue delay. Hence, we can predict that, for the single report condition, at least one of the standard deviations of the Gaussians should vary with the cue delay.

*Model D*: Considering selective vs. nonselective transfer, these two mechanisms may imply different characteristics for items stored in STM; for example, higher precision may be allocated to the items transferred selectively than nonselectively. To distinguish two different kinds of information transfer, we formulated Model D (Figure 3D). Model D permits a distinction among items stored in STM according to the type of transfer they underwent. The model can be described as:

(9) $$\begin{array}{c} S_{R}\left(\varepsilon \right)=\omega_{1}G_{SM}\left({\varepsilon ;\mu }_{SM1},{\sigma_{SM1}}^{2}\left(t\right)\right)\;*\;G_{STM}\left(\varepsilon ;\mu_{STM1},{\sigma_{STM1}}^{2}\right)+\\ \;\omega_{2}G_{SM}\left(\varepsilon ;\mu_{SM2},{\sigma_{SM2}}^{2}\left(t\right)\right)\;*\;G_{STM}\left(\varepsilon ;\mu_{STM2},{\sigma_{STM2}}^{2}\right)+\left(1-\omega_{1}-\omega_{2}\right)U\left(a,b\right) \end{array}$$

By simplifying, we obtain:

(10) $$\begin{array}{c} S_{R}\left(\varepsilon \right)=\omega_{1}G_{Memory1}\left({\varepsilon ;\mu }_{SM1}+\mu_{STM1},{\sigma_{SM1}}^{2}\left(t\right)+{\sigma_{STM1}}^{2}\right)+\\ \;\omega_{2}G_{Memory2}\left({\varepsilon ;\mu }_{SM2}+\mu_{STM2},{\sigma_{SM2}}^{2}\left(t\right)+{\sigma_{STM2}}^{2}\right)+\left(1-\omega_{1}-\omega_{2}\right)U\left(a,b\right) \end{array}$$

For the full report condition, all disks were cued for reporting, and the first full report item was from STM, which was transferred nonselectively before the cue appeared. However, for the single report condition, one of the three disks was randomly selected. The direction for report could be from nonselective transfer, such as the first full report, or from selective transfer. Two Gaussians captured the selective and nonselective transfer, respectively. Since the first full report (FR1) would come from nonselective transfer, we predicted that the one Gaussian + uniform model would capture data better for this report condition. However, we expected a single report to involve nonselective transfer and selective transfer, which means the two Gaussians + uniform mixture model should capture the data better for the single report condition.

Even though the simplified results for both Model C and Model D showed that they were two Gaussians + uniform mixture models, the signatures of those two models were different, and we can easily distinguish between those two models if the two Gaussians + uniform mixture model turns out to be the best one.

## 3. Results

### 3.1. Testing the Leaky Flask Model on the New Dataset

As mentioned in the Introduction, the leaky flask model was derived based on one dataset. One goal of the current study was to test if the results held for an alternative dataset. The modeling of the original data was performed by using a mixture model consisting of one Gaussian and a uniform distribution. Hence, our first step was to replicate the same approach by using a different dataset and assess if there was agreement between the modeling results across the two different datasets.

In Experiment 1, the cue delay was set to zero to minimize the involvement of memory processing. The stimuli consisted of one, two, three, or four moving disks for which the direction of motion was to be reported. Among the different report types, there were two kinds that were of particular interest for this part of the study: single report (SR), i.e., the one item randomly selected by the computer for reporting; the first item in the full report condition (FR1), i.e., the first item that the participant chose to report in each trial. The statistics showed that SR and FR1 were not significantly different across set size $\left(F=0.772,p=0.444,\eta_{P}^{2}=0.205\right)$ (from Tripathy and Öğmen [22], Table 1, Line 14), which means the involvement of memory capacity was limited or negligible, as one would expect from a cue delay of zero. Presenting the cue immediately after the termination of motion ensured that the reporting performance reflected the pre-memory early encoding stage of motion processing.

**Figure 4:**
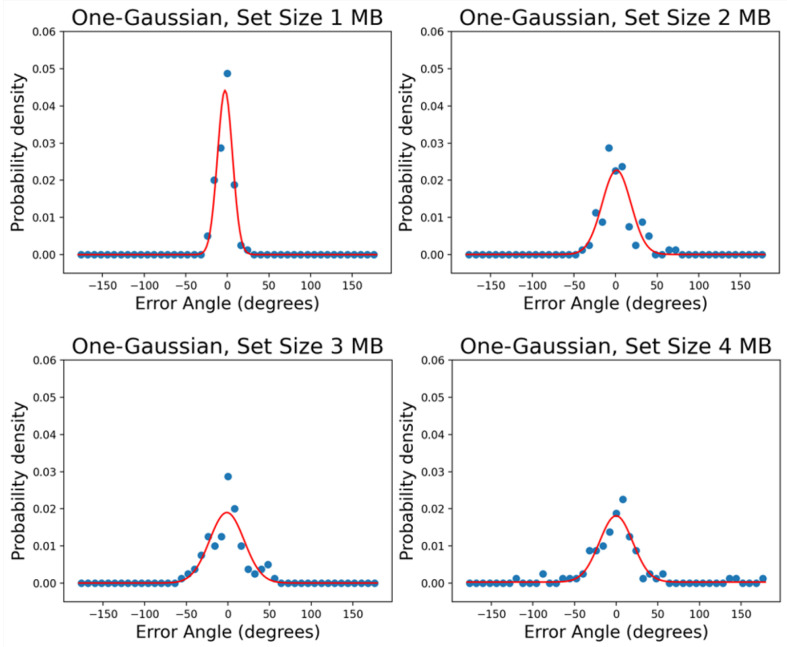
An example of the one Gaussian + uniform mixture model fits for the single report condition. The probability density is shown as a function of the error angle. The figures show the data from subject MB. The dots are the data with a bin size of $4^{\circ }$. The red solid curves are the probability density functions that were replotted from the cumulative distribution fitting.

**Figure 5:**
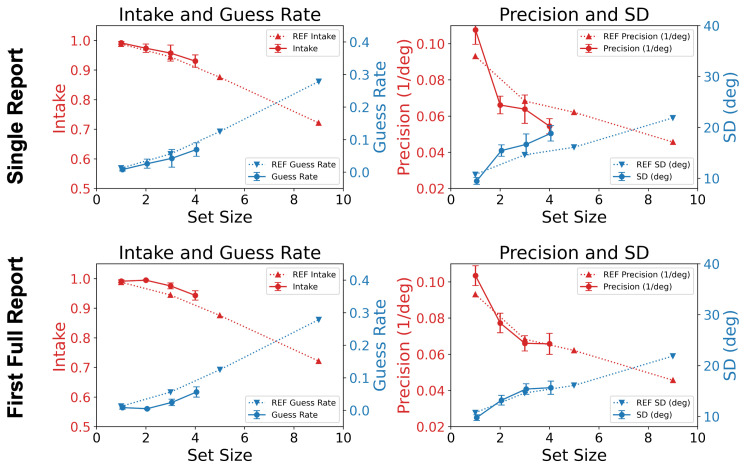
Intake (**left column**) and precision (**right column**) as a function of set size. Error bars represent ±SEM. The plots also include the guess rate (1−ω) and SD (σ) on the right vertical axes. The solid curves show the results from the data analyzed in the current paper, whereas the dotted curves were replotted from Öğmen et al. [21], abbreviated as REF in the inset of the figure for comparison. Overall, there was good agreement between the results obtained from the two different datasets.

Figure 4 is an example plot of the one Gaussian + uniform mixture model fitting result. The figures show the data from subject MB with different set sizes. The intake and precision results are plotted in Figure 5. The dotted curves replot the data from Öğmen et al. [21]. The solid curves represent the data from the present study.

In the mixture model, μ represents the accuracy and 1/σ the precision of encoding, where σ is the standard deviation. ω is the proportion of trials in which the response is based on the representation of the target, and (1−ω) is the proportion of trials in which the response is based on a guess. The variable ω is a relative measure for the intake of encoding, i.e., the proportion of the items in each trial that are available for reporting, on average, at the encoding stage of processing. Due to the existence of memory and attentional capacity limits in early visual processing, we expected that the intake and precision parameters would decrease with increasing set size. Figure 5 shows different drop rates in the intake and precision for both SR and FR1. To capture this observation, we separated the characterizations of intake and precision in our model.

**Figure 6:**
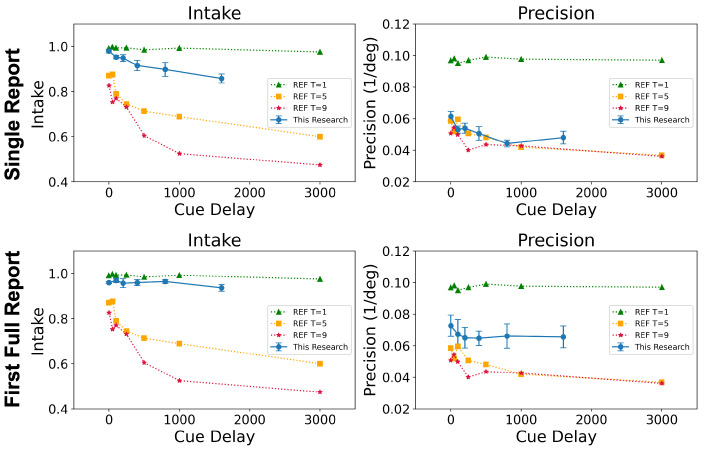
Intake (**left column**) and precision (**right column**) as a function of cue delay. Error bars represent ±SEM. Solid curves are from the current study (target set size = 3). The dotted curves (the target set sizes were set to 1, 5, and 9) are from Öğmen et al. [21], abbreviated as REF in the inset for comparison.

In Experiment 2, the set size was fixed at three with the cue delay being set to one of the following values: 0 ms, 100 ms, 200 ms, 400 ms, 800 ms, or 1600 ms. As in Experiment 1, the reporting conditions included the SR and FR1 conditions among other reporting conditions that were not modeled here. Introducing non-zero cue delays in the two different reporting conditions resulted in the involvement of the memory stages of processing. The transformed performances of SR and FR1 were matched at the encoding stage (cue delay = 0 ms), but were significantly different from each other as the cue delay increased. In Figure 6, the dashed curves plot the data from Öğmen et al. [21] with target sizes of one (green), five (orange), and nine (red). For the data analyzed in the current study, the set size was three. For this set size, the performance was predicted to lie between the performance for set sizes of one (green) and five (orange). For SR (solid blue curve), we considered a target size of one in Öğmen et al.’s paper [21] as the baseline. The quality of information (precision) mainly dropped at the stimulus encoding stage (i.e., cue delay = 0 ms) and remained relatively unchanged with longer cue delays, which indicates a bottleneck in processing at the encoding stage before information starts to decay in SM. The drop in the quantity of information (intake) was more distributed, spreading across the stimulus encoding, SM, and STM stages.

Since in condition FR1, the subjects could always choose to report their best-remembered item, the effect of the cue delay on intake was not as significant as in the SR condition. However, the results still supported the prediction that the quality of information (precision) encounters a bottleneck at the early stage of stimulus encoding. Both SR and FR1 clearly showed major bottlenecks, i.e., steep precision decreases for a set size of three compared to a set size of one, occurring at the early stage of stimulus encoding. On the other hand, the quantity of information (intake) gradually decreased across the encoding and memory stages.

In summary, in this section, we compared the results from our earlier study leading to the formulation of the leaky flask model to the results obtained by analyzing data from a different study. The good agreement between the results obtained from the two different datasets provided evidence for the generality of the leaky flask model.

### 3.2. Multiple Gaussians Models

Given the complexity of the information processing stages involved in the encoding and storage of information and considering recent studies proposing variable precision in STM [26,33,34,35], we questioned whether mixture models with more than one Gaussian component can account better for the data and whether an infinite number of Gaussians is necessary, as implied in the variable precision model. Hence, we tested models containing up to five Gaussians. Five hypothetical models are also proposed to address the origin of the variability in memory precision (see Figure 3).

**Figure 7:**
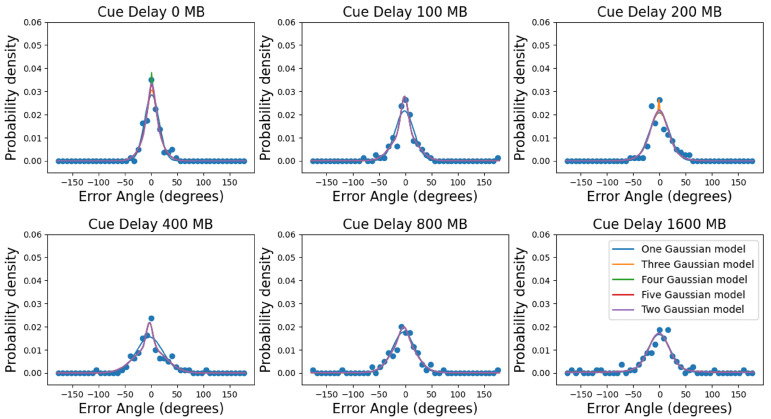
Multiple Gaussian model fitting results. The probability density is plotted as a function of the error angle. Figures show the data of subject MB for different cue delays for the single report condition in Experiment 2. The dots are data with a bin size of 4°. The solid curves are probability density functions that were replotted from the cumulative distribution fitting. The different color solid lines correspond to different mixture models, respectively.

For both Experiment 1 and Experiment 2, we fit the results using the models discussed in the Methods Section. We fit all set size conditions in Experiment 1 and cue delay conditions in Experiment 2 for every single subject (see Figure 7 for an example, subject: MB, single report in Experiment 2). As previous studies [26,33,34,35], here as well, the error distributions appeared to be more “peaked” than a one Gaussian + uniform mixture model would predict (see Figure 7). On the other hand, the two Gaussians + uniform mixture model was able to capture the data very well.

**Figure 8:**
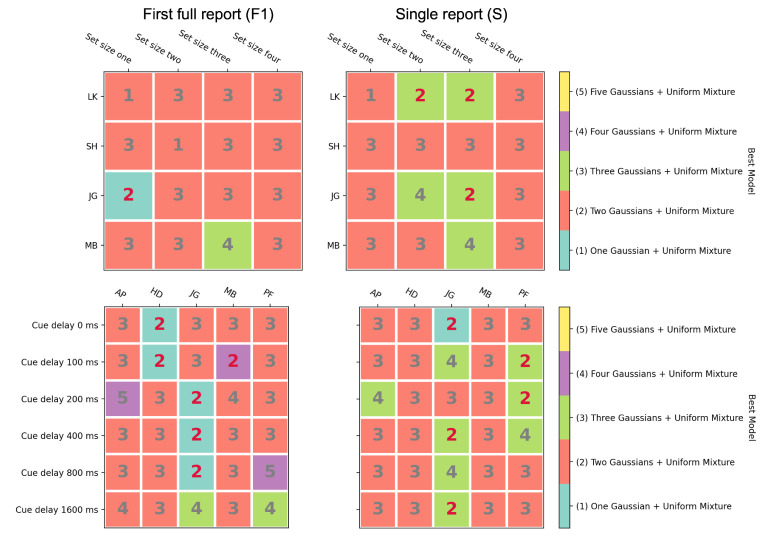
Model comparison results for Experiment 1 (set size) are shown in the top row and results for Experiment 2 (cue delay) in the bottom row. The first full report conditions are in the left column, and the single report conditions are in the right column. The different colors represent different models. The best-fitting model for each condition is displayed in the color map. The number in the color map shows the second-best fitting model for each condition. The two Gaussians + uniform mixture models are also highlighted in red.

The model comparison results are plotted in Figure 8 with different colors representing different models. In each condition, the best-fitting model (smallest BIC value) is shown by the color map. The number in the color map shows the second-best fitting model (e.g., “2” represents the two Gaussians + uniform mixture model). The results clearly showed the two Gaussians + uniform mixture model to be the best model for most of the conditions, which means that Model C or Model D is the model that can best describe the memory processes. For Model D, we had the hypothesis that, in the first full report condition (FR1), subjects would report the best-remembered direction, which should come from nonselective transfer. However, for the single report condition, the disk was randomly selected, which could be from nonselective or selective transfer. Due to these differences, we predicted the one Gaussian + uniform model would be the better model for the first full report condition. Meanwhile, the two Gaussians + uniform mixture model would capture the data better for the single report condition. However, that was not the case. For all conditions, two Gaussians + uniform mixture model could capture the data better. What is more, the two Gaussians + uniform mixture model also supported Model C. Similarly, for the first full report condition, subjects would select the best of the remembered directions to report, which is likely to correspond to one of the items transferred to STM before the cue appeared. In STM, the standard deviation should not vary with the cue delay. For the single report condition, the cued disk may not be the one that was best remembered in STM. When the cue appeared, the relevant information in SM was transferred to the STM for the final report. With cue delay, the performance of SM decayed [1,2,4,21,36,37,43,63]. We can predict that at least one of the standard deviations from the two Gaussians would represent SM and should vary with the cue delay; more specifically, it should increase when the cue delay increases.

**Figure 9:**
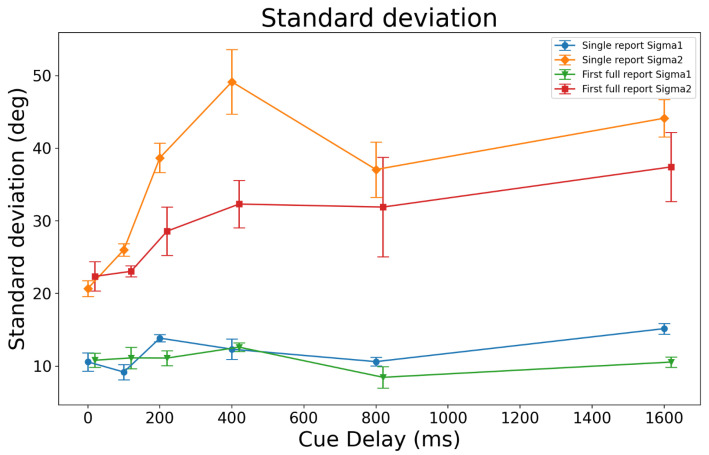
Standard deviations averaged across observers as a function of the cue delay. Error bars represent ±SEM. Of the two standard deviation parameters, the one with the smaller value is independent of the cue delay in both the single report and first full report conditions. For the standard deviation with the larger value, its dependence on the cue delay did not reach significance for the FR1 condition, whereas it did for the SR condition.

Thus, a key prediction of the models concerns the dependence of the standard deviations on the cue delay. Figure 9 plots the standard deviations of the two Gaussians in the model as a function of the cue delay for the SR and FR1 conditions. In these plots and the corresponding analyses, the averaged values of the standard deviations across observers were used. The inspection of Figure 9 indicates that the standard deviation with a lower value (single report Sigma1 and first full report Sigma1 in Figure 9) was largely independent of the cue delay for both the SR and FR1 conditions, as confirmed by the RM-ANOVA results (F(5,20)=1.031,p=0.426 and F(5,20)=0.476,p=0.789), for SR and FR1, respectively.

For the FR1 condition, the standard deviation with a larger value (first full report Sigma2 in Figure 9) did not depend on the cue delay either, although there seemed to be a tendency to increase (F(5,20)=0.600,p=0.701). The results supported our prediction that at least one of the two standard deviation parameters would not vary with the cue delay. However, for the SR condition, the standard deviation with the larger value (single report Sigma2 in Figure 9) showed a significant increase with the cue delay (F(5,20)=3.197, p=0.028). These results supported the prediction that one of the standard deviations varied with the cue delay. In summary, all these results supported Model C.

## 4. Discussion

### 4.1. Confirmation of the Leaky Flask Model

STM plays a very important role in our daily lives. Due to its limited capacity [5,6,7,8,9,10,11,12,13,14,15,16,17,18], storing and updating the information in STM at the proper time is crucial for the mental process [9,10,19,20]. The commonly accepted view is that the major bottleneck to information processing is in STM, as illustrated by the leaky hourglass model illustrated in Figure 1. Contrary to this view, Ogmen et al. [21] showed that the major bottlenecks for motion processing exist prior to STM, as illustrated by their proposed leaky flask model [21]. We tested this new model by using data for processing motion direction from a novel psychophysical study [22]. We followed the methods of Öğmen et al. [21] to estimate qualitative and quantitative measures of performance for the dataset in Tripathy and Öğmen [22]. As Figure 5 shows, very good agreement of the effect of the set size on intake and precision across the two studies was found, in particular in the first full report condition. Figure 6 also shows a very good agreement for the effect of cue delay on intake and precision across the two studies. More importantly, consistent with the earlier study, the quality of information (precision) mainly dropped at the stimulus encoding stage (cue delay = 0 ms) and remained relatively unchanged with longer cue delays, which indicated a bottleneck in processing at the encoding stage before information started to decay in SM. The drop in the quantity of information (intake) was more distributed, spreading across the stimulus encoding, SM, and STM stages. These new results provide additional empirical support for the leaky flask model indicated by the data from a very different experiment in an earlier study. The two studies highlight the existence of very significant bottlenecks at the encoding stage, prior to processing at the SM stage.

### 4.2. A Few Gaussians Are Adequate to Accurately Model the Data for Recall of Direction of Motion

Several recent studies have proposed that the recall of information such as color can be modeled as a combination of an infinite number of Gaussian distributions, i.e., as a gamma distribution [26,30,33,34,35]. These studies were motivated by the fact that the distribution of the data from some of the recall studies was more peaked than the standard Gaussian distribution. We also found that our distributions for recalled information of the direction of motion deviated from the normal distribution and questioned whether models using a combination of a small number of Gaussians could fit our data adequately. We used statistical mixture models of the n-Gaussians + uniform (n = 1,2,…,5) type to fit the data from Tripathy and Öğmen [22] and compared the quality of the fits (smallest BIC score) for the different values of n. One would predict, based on the earlier studies, that the quality of the fits to the data would improve as n increased and the best fits to the data would result from combining a greater number of Gaussians [26,30,33,34,35]. Contrary to this prediction, we found that the data for a majority of conditions (66 out of the 92 color squares in Figure 8) was best modeled by the two Gaussians + uniform mixture (Figure 8). In the conditions where the two Gaussians + uniform model was not the best fit to the data, it was frequently the second best (15 out of 26), as can be seen from the boxes labeled with a red 2 in Figure 8. In only two of the ninety-two cases was the five Gaussians + uniform mixture the winner or runner-up in terms of the quality of the fit to the data. This indicates that the direction of motion data from the Tripathy and Öğmen [22] study can be optimally modeled by as few as two Gaussians and that neither a large, nor infinite number of Gaussians is required to model these data.

### 4.3. Modeling Different Configurations of Storage and Recall

Figure 3 shows proposed block diagrams for four different configurations (Models A–D) of memory and attention. The associated Equations (3)–(10) show that Models A and B fall under the one Gaussian + uniform category, and Models C and D fall under the two Gaussians + uniform mixture model category. As shown in Figure 8, and summarized in the previous section, our modeling suggests that the two Gaussians + uniform mixture model best supports the direction of motion data in Tripathy and Öğmen [22]. Of the configurations proposed in Figure 3, Models C and D yield equations that are consistent with the two Gaussians + uniform mixture model. In both of these configurations, attention plays an important role, represented either explicitly as a block in Model C or implicitly in the selective transfer between SM and STM in Model D. The two models can be distinguished based on the equations representing the models. The mathematical representation of Model C (Equation (8)) predicts that of the two Gaussians in the mixture, one would have an SD that is a function of time, whereas the other SD would be independent of time (on the timescale of the duration of each trial). The mathematical representation of Model D (Equation (10)) predicted that both Gaussians would have SDs that are functions of time. The data in Figure 9 favored Model C over Model D; Sigma1 for SR and FR1 were essentially unaffected by the cue delays of up to 1600 ms, and Sigma2 for both SR and FR1 increased as the cue delay increased over the same range. Equation (8) and Figure 9 together give us insights into the relative precisions of the different blocks in Model C—the precision of attention and STM remained high (SD was low) and stable over time, whereas that of SM decreased over time.

### 4.4. Limitations of the Current Study

The current study attempted to model only a subset of the conditions for which data were collected in the Tripathy and Öğmen [22] study. The earlier study collected the information of direction for the pre-deviation parts and the post-deviation parts of the trajectories. The study found that event segmentation from the synchronized change in direction of the moving disks resulted in sensory memory being preferentially allocated to the post-deviation event segment compared to the pre-deviation event segment [64,65]. The current study did not attempt to model event segmentation and did not attempt to model the pre-deviation segments of the trajectories. Modeling event segments prior to the last event segment would require making assumptions relating to the distribution of attention and memory across event segments, which have not been incorporated into the current model. The stimulus in the post-deviation event segment in the Tripathy and Öğmen [22] study is comparable to the stimuli in Öğmen et al. [21], permitting the current study to adapt, and to elaborate, the modeling approach used in the earlier study.

In the FR condition in the Tripathy and Öğmen [22] study, observers reported each of the directions of motion within an event segment. In the cue delay experiment, each trial requiring FR would require three directions of motion to be reported. Of these, only the first reported response in each trial was modeled. Further elaboration of the model is required to address the issue of 2nd, 3rd, …, nth response in an FR trial. Thus, the modeling in the current study did not capture the full richness of the data in Tripathy and Öğmen [22]. This study took a few initial steps for modeling the direction of motion. It showed that the direction of motion can be modeled by mixture models using a limited number of Gaussians and that a configuration similar to Model C in Figure 3 is a good starting point. However, the models proposed here have to be elaborated further to deal with event segmentation and multiple reports during FR. Future work will attempt to address these shortcomings.

Figure 3 proposes block diagrams for a set of plausible configurations for memory and attention. This set is not exhaustive, and other configurations can be proposed that are plausible. However, our finding that a mixture model of the form of the two Gaussians + uniform mixture model best represents the direction of motion data would constrain any alternative configuration proposed.

We did not specifically model interference in the current study. In the previous studies [21,36], we investigated a form of interference by including “misbinding” components in our models. When two items are similar to each other, they may interfere with storage and retrieval, and “misbinding’ refers to incorrectly reporting an interfering item. In the study of Ögmen et al. [21], the models that included misbinding components were not the ones that best captured the data. In the study of Huynh et al. [36], the misbinding components were very small, especially in the small dataset conditions. Furthermore, the experiment modeled in this study and the previous related experiments were designed to minimize the influence of similarity-based interference by ensuring that no direction of motion represented in a trial was close to the other directions present. In the study of Huynh et al. [36], all directions represented were separated at least by 17${}^{\circ }$. In the data used here, the directions were separated by more than 20${}^{\circ }$. Another form of interference may come from items that were stored earlier in a sequence. For example, Gorgoraptis et al. [32] and Zokaei et al. [66] showed that the errors associated with the last items in a sequence were mainly the result of an increase in variability in memory. Hence, interference should be included in a more general version of this model in order to apply it to more realistic cases that include similar items learned in a sequential regime.

Another missing component of this model is the effect of rehearsal. For example, the Baddeley–Hitch model includes a phonological loop, which allows articulatory rehearsal so as to prolong the maintenance of information in working memory [67]. Rehearsal is most effective when information can be expressed phonologically, for example when recalling discrete symbols such as letters and digits. It is harder and less relevant for the complex stimulus patterns when reporting directions of motion.

Finally, let us highlight that the models and tests conducted in this and our previous studies examined how motion direction information is stored in memory. Similar studies using other stimulus characteristics, such as shape, color, etc., need to be conducted to test the generality of the model. 

## Data Availability

Data are available upon request from the authors.

## Abbreviations

The following abbreviations are used in this manuscript:

MDPI	Multidisciplinary Digital Publishing Institute
DOAJ	Directory of open access journals
TLA	Three letter acronym
LD	Linear dichroism

## Appendix A. Fitting Methods

We modeled the data by varying the number of Gaussians within an n-Gaussians + uniform mixture model. We tested how many Gaussians were needed in the mixture model to best capture the directions reported in the first full report and single report conditions. We tested up to five Gaussians for the mixture model. The Bayesian information criterion (BIC) [68] was used to determine the number of Gaussians needed to best model the direction of motion data. After that, we further analyzed the best configuration of the components in terms of Models A–D discussed above.

For the model fitting part, to avoid issues related to binning, we used the cumulative equations for fitting. The fitting was performed with Python using the package LMFIT [69]. After fitting, the probability density curves were obtained from the cumulative distributions. To display the results for the probability density plots, the data were binned with intervals of size $4^{\circ }$. The cumulative equation used for one Gaussian + uniform mixture model was:

(A1) $${CDF}_{1G+U}\left(\varepsilon \right)=\omega_{1}\phi_{1}\left(\varepsilon ;\mu_{1},\sigma_{1}\right)+\left(1-\omega_{1}\right)\frac{\varepsilon -\left(-180\right)}{180-\left(-180\right)}$$

where ϕ is the cumulative Gaussian distribution, the second term in the equation is the cumulative uniform distribution, and the sum of the weights of the terms was constrained to one. Higher-order cumulative distributions were recursively defined by the following equations. The fitting results were reformatted and shown using the equations in the Model Section. The cumulative equation for the two Gaussians + uniform mixture model:

(A2) $${CDF}_{2G+U}\left(\varepsilon \right)=\omega_{2}\phi_{2}\left(\varepsilon ;\mu_{2},\sigma_{2}\right)+\left(1-\omega_{2}\right){CDF}_{1G+U}\left(\varepsilon \right)$$

The cumulative equation for the three Gaussians + uniform mixture model:

(A3) $${CDF}_{3G+U}\left(\varepsilon \right)=\omega_{3}\phi_{3}\left(\varepsilon ;\mu_{3},\sigma_{3}\right)+\left(1-\omega_{3}\right){CDF}_{2G+U}\left(\varepsilon \right)$$

The cumulative equation for the four Gaussians + uniform mixture model:

(A4) $${CDF}_{4G+U}\left(\varepsilon \right)=\omega_{4}\phi_{4}\left(\varepsilon ;\mu_{4},\sigma_{4}\right)+\left(1-\omega_{4}\right){CDF}_{3G+U}\left(\varepsilon \right)$$

The cumulative equation for the five Gaussians + uniform mixture model:

(A5) $${CDF}_{5G+U}\left(\varepsilon \right)=\omega_{5}\phi_{5}\left(\varepsilon ;\mu_{5},\sigma_{5}\right)+\left(1-\omega_{5}\right){CDF}_{4G+U}\left(\varepsilon \right)$$

Correspondingly, the probability density functions that were used to plot the probability density distribution are listed below. The one Gaussian + uniform mixture model:

(A6) $${PDF}_{1G+U}\left(\varepsilon \right)=\omega_{1}G_{1}\left(\varepsilon ;\mu_{1},\sigma_{1}\right)+\left(1-\omega_{1}\right)U\left(-180,180\right)$$

The two Gaussians + uniform mixture model:

(A7) $${PDF}_{2G+U}\left(\varepsilon \right)=\omega_{2}G_{2}\left(\varepsilon ;\mu_{2},\sigma_{2}\right)+\left(1-\omega_{2}\right){PDF}_{1G+U}\left(\varepsilon \right)$$

The three Gaussians + uniform mixture model:

(A8) $${PDF}_{3G+U}\left(\varepsilon \right)=\omega_{3}G_{3}\left(\varepsilon ;\mu_{3},\sigma_{3}\right)+\left(1-\omega_{3}\right){PDF}_{2G+U}\left(\varepsilon \right)$$

The four Gaussians + uniform mixture model:

(A9) $${PDF}_{4G+U}\left(\varepsilon \right)=\omega_{4}G_{4}\left(\varepsilon ;\mu_{4},\sigma_{4}\right)+\left(1-\omega_{4}\right){PDF}_{3G+U}\left(\varepsilon \right)$$

The five Gaussians + uniform mixture model:

(A10) $${PDF}_{5G+U}\left(\varepsilon \right)=\omega_{5}G_{5}\left(\varepsilon ;\mu_{5},\sigma_{5}\right)+\left(1-\omega_{5}\right){PDF}_{4G+U}\left(\varepsilon \right)$$

The BIC was used for the model comparison:

(A11) $$BIC=Kln\left(n\right)-2ln\left(\hat{L}\right)$$

where *K* is the number of estimated parameters in the model and *n* is the number of observations. $\hat{L}$ is the maximum value of the likelihood function for the model. A smaller BIC means a better fit. The BIC was calculated using the Python package RegscorePy.
